# Structural heterogeneity of *attC* integron recombination sites revealed by optical tweezers

**DOI:** 10.1093/nar/gky1258

**Published:** 2018-12-19

**Authors:** Ann Mukhortava, Matthias Pöge, Maj Svea Grieb, Aleksandra Nivina, Celine Loot, Didier Mazel, Michael Schlierf

**Affiliations:** 1 B CUBE – Center for Molecular Bioengineering, TU Dresden, Tatzberg 41, 01307 Dresden, Germany; 2 Institut Pasteur, Unité de Plasticité du Génome Bactérien, Département Génomes et Génétique, 28 Rue du Dr Roux, 75015 Paris, France; 3 CNRS, UMR3525, 28 Rue du Dr Roux, 75015 Paris, France; 4 Paris Descartes University, 75006 Paris, France

## Abstract

A predominant tool for adaptation in Gram-negative bacteria is the functional genetic platform called integron. Integrons capture and rearrange promoterless gene cassettes in a unique recombination process involving the recognition of folded single-stranded DNA hairpins—so-called *attC* sites—with a strong preference for the *attC* bottom strand. While structural elements have been identified to promote this preference, their mechanistic action remains incomplete. Here, we used high-resolution single-molecule optical tweezers (OT) to characterize secondary structures formed by the *attC* bottom (${{att}}{{{C}}_{{\rm{bs}}}}$) and top (${{att}}{{{C}}_{{\rm{ts}}}}$) strands of the paradigmatic *attC_aadA7_* site. We found for both sequences two structures—a straight, canonical hairpin and a kinked hairpin. Remarkably, the recombination-preferred ${{att}}{{{C}}_{{\rm{bs}}}}$ predominantly formed the straight hairpin, while the ${{att}}{{{C}}_{{\rm{ts}}}}$ preferentially adopted the kinked structure, which exposes only one complete recombinase binding box. By a mutational analysis, we identified three bases in the unpaired central spacer, which could invert the preferred conformations and increase the recombination frequency of the ${{att}}{{{C}}_{{\rm{ts}}}}$*in vivo*. A bioinformatics screen revealed structural bias toward a straight, canonical hairpin conformation in the bottom strand of many antibiotic resistance cassettes *attC* sites. Thus, we anticipate that structural fine tuning could be a mechanism in many biologically active DNA hairpins.

## INTRODUCTION

**Figure 1.**
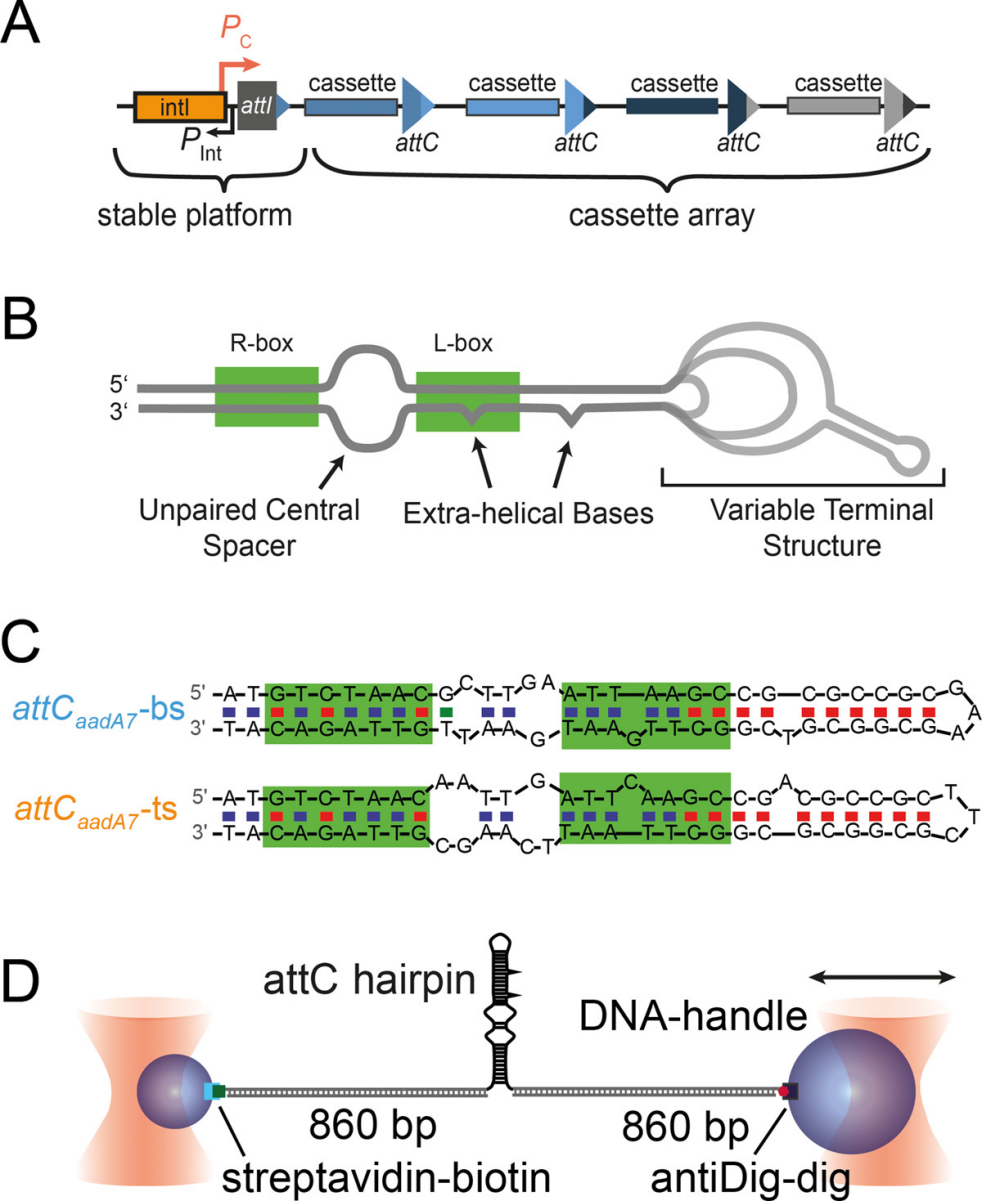
The integron system and schematic structure of *attC* hairpins. (**A**) Schematic depiction of the integron stable platform and cassette array. (**B**) Schematic of the structure of *attC* sites. (**C**) Canonical structure prediction of *attC_aadA7_* bottom and top strand. **(D)** Optical tweezers assay to study DNA hairpin secondary structure by mechanical unfolding and refolding.

Bacteria possess an impressive capability of adapting to environmental stresses. They are known to develop antibiotic multi-resistance by horizontal gene transfer. The predominant tool for the acquisition and expression of resistance genes in Gram-negative pathogens is a genetic device called integron (1,2). Integrons capture and rearrange promoterless gene cassettes in a unique recombination process involving the excision and integration of single-stranded DNA. Upon SOS response, the integron tyrosine-recombinase IntI specifically recognizes folded, single-stranded DNA hairpins called *attC* sites, which flank the open reading frame in cassettes (3,4) (Figure 1A). IntI excises gene cassettes by recombination between two consecutive *attC* sites and integrates the cassettes predominately at the *attI* site. To ensure correct expression of the protein-coding sequences from these cassettes, the orientation of the inserted DNA sequence has to be tightly controlled with respect to the cassette promoter *P*_c_. Bouvier *et al.* and Nivina *et al.* have identified important structural elements of *attC* sites, which ensure the correct orientation through the strand-selectivity of IntI for the bottom strand (${{att}}{{{C}}_{{\rm{bs}}}}$) relative to the top strand (${{att}}{{{C}}_{{\rm{ts}}}}$) (5,6). Those structural elements are extra-helical bases (EHBs), an unpaired central spacer (UCS) and a variable terminal structure (VTS, Figure 1B). The role of the EHBs could be inferred from a crystallographic structure due to their prominent interactions with the recombinase IntI (7) and was further shown to be the main factor determining the strand specificity with a contribution of 20 to 300-fold in the strand recognition, depending on the site tested (5,6). The mechanisms imposed by the UCS and the VTS remain unclear. A recent study has reported that a nucleotide skew induced by UCS and VTS stabilizes the folded bottom strand, and by that most likely influences the recombination indirectly (6). An earlier study has suggested that *attC* bottom strands recombine less efficiently if stable parasitic non-recombinogenic secondary structures are formed (8). However, an experimental structural comparison of *attC* bottom strands and top strands has not been carried out to date, despite the apparent importance of the secondary structure of *attC* hairpins for the recombination by IntI.

Here, we used high-resolution single-molecule optical tweezers to characterize secondary structures formed by bottom strand (${{att}}{{{C}}_{{\rm{bs}}}}$) and top strand (${{att}}{{{C}}_{{\rm{ts}}}}$) of *attC_aadA7_* site (Figure 1C, D). The force-induced unfolding revealed the existence of two structurally distinct, but energetically similar conformations for both hairpins. The predominant conformation of *attC*_bs_ exposed the IntI binding site, while the predominant conformation of *attC*_ts_ partially buried the binding site. The comparison between wild-type and mutant sequences yielded important insights into the regulation of the exposure of the integrase binding site by a few nucleotides within the *attC_aadA7_* hairpin. This is directly linked to the efficiency of recombination and thus we speculate that this regulation mechanism is important for strand selectivity as we found a conservation in a bioinformatics analysis of 263 known *attC* sites.

## MATERIALS AND METHODS

### Hairpin sequences

All hairpin sequences were flanked with overhangs of 20 bases on each side complimentary to the DNA handles and a 3 dT spacer. The hairpins were inserted between two double-stranded handles of ≈860 base pairs (bp) each (Figure 1D). Biotin- and digoxigenin-functionalized 852 bp (290 nm) and 855 bp (291 nm) DNA handles were synthesized by PCR amplification of λ-DNA. The 5′ ends of the forward primers were functionalized with biotin or digoxigenin. The reverse primers introduced nicking enzyme sites to generate a 20-nucleotide ssDNA overhang complementary to the hairpin extensions. Subsequent to PCR amplification, the biotin- and the digoxigenin-handles were purified by ethanol precipitation and then digested with the nicking enzymes Nb.BsmI and Nb.BsrDI, respectively. All oligonucleotides were purchased from Sigma-Aldrich Co. To hybridize the handles with the hairpin, a 1.5:1:1 mixture of the hairpin and DNA handles, respectively, was incubated for 2 min at 90°C. Then the sample was slowly cooled down to 16°C and further ligated 12 h at 16°C with T4 DNA ligase (New England Biolabs). The final product was purified by ethanol precipitation. The full sequences of the DNA hairpins and primers for the handle construction are shown in the Supplementary Table S1A.

### Single-molecule optical tweezers experiments

DNA constructs were incubated for 2 h with 2 μm silica beads (Bangs Laboratories, Inc), which were previously covalently functionalized with anti-digoxigenin F_ab_ fragments (Roche). The mixture was diluted in optical tweezers buffer (50 mM sodium phosphate buffer, 250 mM NaCl, 1% (w/v) d-glucose, pH 7.1) and mixed with streptavidin-coated 1 μm silica beads (Bangs Laboratories, Inc.). Measurements were carried out at room temperature in optical tweezers buffer after addition of an oxygen scavenger system (26 U/ml glucose oxidase, 17 000 U/ml catalase). DNA conjugates concentration was adjusted to only sparsely cover the beads leading mainly to single-tether formation. The beads were trapped in the foci of a dual beam optical tweezers platform (JPK NanoTracker) (9). Both trapped beads were brought into close proximity for tether formation. The beads were then separated with a constant velocity yielding force *vs*. extension traces. Trap stiffness was determined using a previously described calibration protocol (10). The effective trap stiffness was determined with an error of ∼10% and varied from 0.056 to 0.062 pN/nm. Data were acquired at a sampling rate of 50 kHz. The signals were corrected for crosstalk.

### Force-extension curves and contour length increase

The recorded force–extension curves can be well described with polymer elasticity models. Before unfolding, the DNA elasticity was modeled with the worm-like chain model (WLC) for the double-stranded DNA handles (11,12):

\begin{eqnarray*}{F_{{\rm{WL}}{{\rm{C}}_{{\rm{handles}}}}}}\ \left( x \right) &=& \frac{{{k_{\rm{B}}}T}}{{{p_{{\rm{dsDNA}}}}}}\nonumber\\ &&\times\,\left( {\frac{1}{4}{{\left( {1 - \frac{x}{{{L_{{\rm{handles}}}}}}} \right)}^{ - 2}} + \frac{x}{{{L_{{\rm{handles}}}}}} - \frac{1}{4}} \right),\end{eqnarray*}

with the persistence length ${p_{{\rm{dsDNA}}}}$ and contour length ${L_{{\rm{handles}}}}$ and extension *x*. DNA contour length was set to 580 nm and the fit yielded persistence lengths of ∼20–27 nm. After unfolding of the hairpin, a combined worm-like chain model was used, modeling the single-stranded DNA elasticity with:

\begin{eqnarray*}{F_{{\rm{WL}}{{\rm{C}}_{{\rm{ssDNA}}}}}}\left( {{\xi _{{\rm{hairpin}}}}} \right) &=& \frac{{{k_{\rm{B}}}T}}{{{p_{{\rm{ssDNA}}}}}}\nonumber\\ &&\times\left( {\frac{1}{4}{{\left( {1 \!-\! \frac{{{\xi _{{\rm{hairpin}}}}}}{{{L_{{\rm{ssDNA}}}}}}} \right)}^{\!- 2}} \!+\! \frac{{{\xi _{{\rm{hairpin}}}}}}{{{L_{{\rm{ssDNA}}}}}} \!-\! \frac{1}{4}} \right),\end{eqnarray*}

with persistence length ${p_{{\rm{ssDNA}}}}$ set to 2 nm (13,14), contour length ${L_{{\rm{ssDNA}}}}$, and extension $\xi_{\mathrm{hairpin}}$. Upon force-induced unfolding of the hairpin, a flexible ssDNA chain is added to the compliance of the dsDNA construct. Thus, a combined worm-like chain model was used. As the two mechanical parts consisting of dsDNA and unfolded hairpin (ssDNA) were in series, the extension of the full linker consisting of dsDNA and unfolded hairpin was given by:

\begin{equation*}{\xi _{{\rm{construct}}}}\ \left( F \right) = \ x\left( F \right) + \ {\xi _{{\rm{hairpin}}}}\left( F \right)\end{equation*}

where $x$ and ${\xi _{hairpin}}$ were calculated using the previous equations. This equation was inversed to describe ${F_{{\rm{construct}}}}( {{\xi _{{\rm{hairpin}}}},\ x} )$.

### *In vivo* recombination assay

Wild-type and mutant *attC* sites were constructed by annealing of two overlapping phosphorylated oligonucleotides (Supplementary Table S1D), fully complementary except for the overhangs corresponding to EcoRI and BamHI restriction sites. The sites were then ligated into the p4116 plasmid for the delivery of the bottom strand, and into p4117 plasmid for the delivery of the top strand, both digested with EcoRI/BamHI. The plasmids were then transformed into the *Escherichia coli* β2163 strain (15). The recombination frequency and strand selectivity were determined using a previously developed (16) suicidal conjugation assay described in detail in (6). Briefly, pSW23T vectors were transferred through conjugation from the β2163 strain into a recipient *E. coli* DH5α strain, so that the transferred strand carried either the bottom or the top strand of *attC* sites. The recipient strain harbored the pBAD plasmid allowing the expression of IntI1 integrase, and a pSU plasmid with an *attI1* site. Successful *attI1* × *attC* recombination in the recipient strain conferred its resistance to Cm, allowing us to measure the rate of recombinant cells (Cm^R^) among the total population of recipient cells (Cm^S^) by plating. Recombination frequencies correspond to the average of six independent experiments. For each construct, the recombination frequencies in each strand (bs or ts) was determined by 48 PCR reactions using primer pairs SWbeg/MFD and SWend/MFD as previously described (6). The limits of detection correspond to the minimal frequencies of recombination in either strand that could be detected by this method.

### Bioinformatics analysis of *attC_aadA7_* site and *attC* sites from the INTEGRALL database

We predicted *attC_aadA7_* site conformations using UNAFold (17) under the experimental conditions of our force spectroscopy experiments (0.25 M Na^+^ and 0 M Mg^2+^ ions at 25°C; assumption of a linear DNA molecule). The structures were generated confining the free energies within 5% difference of the minimum free energy. No constraints (like pairing the R’ and R’’, and L’ and L’’ sequences) were used. We further used the database containing sequences of 263 mobile integron *attC* sites having less than a 95% identity, obtained from the authors of the INTEGRALL database (18). The simulation conditions were the same, as described above for the *attC_aadA7_* site. The conformations obtained for each *attC* sequence were categorized into five different groups: (i) straight-complete hairpins with R- and L-boxes fully paired, (ii) straight-incomplete hairpins with paired R-box and not fully paired L-box (e.g. bubble); (iii) kinked-complete hairpins with R- and L-boxes fully paired, (iv) kinked-incomplete hairpins with paired R-box and not fully paired L-box and (v) other structures with unpaired R- and/or L-boxes (see Supplementary Figure S8). Based on the free energy of each conformation, the probability of finding the hairpin in that conformation was calculated using a Boltzmann distribution. *attC* bottom strands formed in 46 ± 3% (±S.D.) of the cases a canonical straight structure, *attC* top strands formed in 36 ± 3% (±S.D.) of the cases a canonical straight structure.

## RESULTS

### Unfolding of *attC_aadA7_* bottom and top strands exposes a mechanically stable intermediate

**Figure 2.**
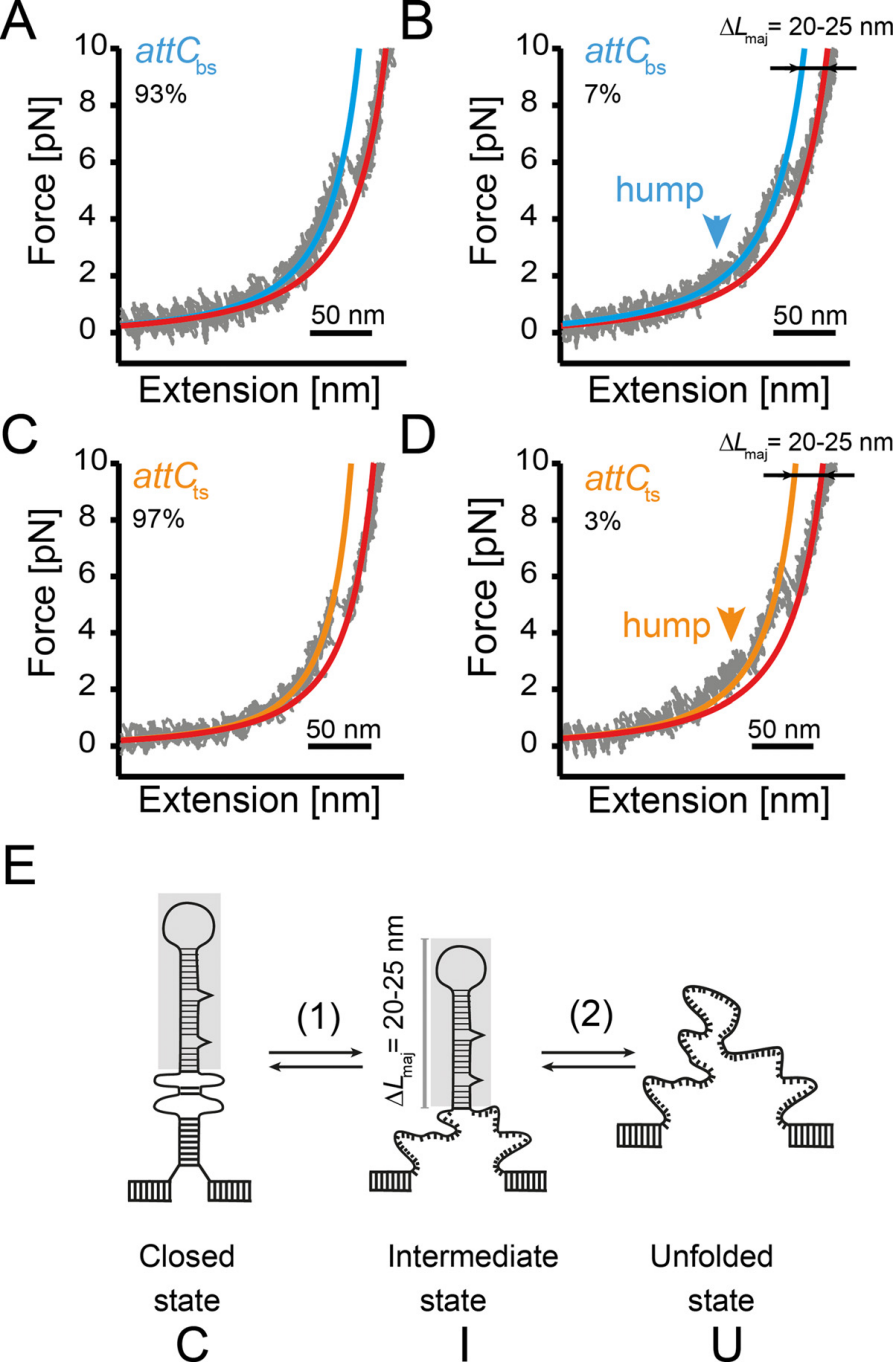
Typical force–extension curves. (**A**) ${{att}}{{{C}}_{{\rm{bs}}}}$ unfolded in 93% of the force–extension curves with one major unfolding event and (**B**) in 7% of the cases with a preceding hump-like unfolding. (**C**) 97% of the ${{att}}{{{C}}_{{\rm{ts}}}}$ force–extension curves show a similar major unfolding event and (**D**) 3% of the traces exhibit a preceding hump-like unfolding. (**E**) Two-step unfolding and refolding of the *attC* hairpin via a mechanically stable intermediate state.

To characterize the secondary structure of the ${{att}}{{{C}}_{{\rm{bs}}}}$ DNA hairpin, it was tethered between two glass microspheres of 1 and 2 μm diameter through two 860-bp-long DNA handles (Figure 1D). By separating the two silica beads from each other, force was applied to the molecular construct, causing the stretching of the DNA handles, which eventually triggered unfolding of the *attC* hairpin. Recording both the force acting on the tether and the positions of beads resulted in force–extension curves (Figure 2). Unfolding events in the force–extension curve contain information about the mechanical stability and the conformation of the DNA hairpin. Figure 2A shows a typical force–extension curve of the ${{att}}{{{C}}_{{\rm{bs}}}}$ hairpin with a constant pulling velocity of 200 nm/s. A sharp transition at a force of approximately 5 pN indicates a structural change, which we attributed to the unfolding of the DNA hairpin structure. The unfolding force of the ${{att}}{{{C}}_{{\rm{bs}}}}$was determined to be *F*_maj_(${{att}}{{{C}}_{{\rm{bs}}}}$) = 5.7 ± 0.8 pN (*k* = 215, mean ± S.D.). In comparison to previous reports on DNA hairpin structures (19) and our supporting measurements on an artificial perfectly-paired hairpin (Supplementary Figure S1), *F*_maj_(${{att}}{{{C}}_{{\rm{bs}}}}$) is surprisingly low. A closer inspection of the force–extension curves of ${{att}}{{{C}}_{{\rm{bs}}}}$ revealed that ≈7% of the traces also contained a second, minor, hump-like unfolding transition at an even lower force *F*_min_(${{att}}{{{C}}_{{\rm{bs}}}}$) ≈ 2.8 ± 0.9 pN (*k* = 15) (Figure 2B), which was stabilized by the addition of 20 mM MgCl_2_ (Supplementary Figure S2). The force at which this minor unfolding event occurred was broadly distributed and often occurred below 1 pN, that prevented this transition to be unambiguously assigned. Our pulling velocity was chosen to be close to equilibrium of the major event, as indicated by multiple un- and refolding events within a single pulling or relaxation cycle (Supplementary Figure S3).

We next measured the contour length change of the major unfolding event (at 5.7 pN) of ${{att}}{{{C}}_{{\rm{bs}}}}$ which allowed us to learn about the underlying structure. The entropic elasticity of dsDNA and ssDNA can be described using a worm-like chain (WLC) polymer elasticity model. Fitting our force–extension traces with a WLC model prior and post unfolding allowed us to extract the contour length change of unfolding the DNA hairpin Δ*L*_maj_ ≈ 20–25 nm. As the ${{att}}{{{C}}_{{\rm{bs}}}}$ contains 64 nucleotides (nt) with a length of 0.68 nm/nt (20) and taking into account a 2 nm end-to-end distance between the 5′- and 3′-ends of the closed DNA hairpin, we would expect a contour length change of approximately 41 nm (64 nt*0.68 nm/nt–2 nm = 41.52 nm) upon full hairpin unfolding. We thus extended the analysis and determined the contour length change occurring during a minor, hump-like unfolding event. The minor unfolding event yielded a contour length change Δ*L*_min_ ≈ 21 nm (Figure 2B). A rigorous analysis of contour length changes during the minor transition with the WLC model remained impossible due to the instability of the fitting algorithm at forces below 2 pN. The sum of the contour length changes of the minor and the major unfolding event Δ*L*_tot_ ≈ 40–44 nm agreed well with the expected contour length increase upon unfolding of the entire ${{att}}{{{C}}_{{\rm{bs}}}}$ hairpin. Therefore, we concluded that the ${{att}}{{{C}}_{{\rm{bs}}}}$ unfolds via a mechanically stable intermediate state that corresponds to a contour length change of Δ*L*_maj_ ≈ 20–25 nm, and, thus, contains ∼30–36 nts (Figure 2E).

In a second set of experiments, we performed the mechanical stretching of the ${{att}}{{{C}}_{{\rm{ts}}}}$ hairpin and observed very similar force–extension curves (Figure 2C, D). The majority of the traces (97%) showed a single unfolding event at *F*_maj_(${{att}}{{{C}}_{{\rm{ts}}}}$) = 5.5 ± 1.0 pN (*k* = 232, mean ± S.D.) with a contour length change of 20–25 nm, similar to ${{att}}{{{C}}_{{\rm{bs}}}}$. Only a few curves (3%) exhibited an additional minor unfolding event at low forces of about *F*_min_(${{att}}{{{C}}_{{\rm{ts}}}}$) = 2.3 ± 0.9 pN (*k* = 8) with a Δ*L*_min_ of about 20 nm. We thus concluded that the ${{att}}{{{C}}_{{\rm{ts}}}}$ unfolds also via a mechanically stable intermediate, like the ${{att}}{{{C}}_{{\rm{bs}}}}$ (Figure 2E).

### ${att}{{C}_{{\rm{bs}}}}$ and ${att}{{C}_{{\rm{ts}}}}$ exist in two distinct conformations

**Figure 3.**
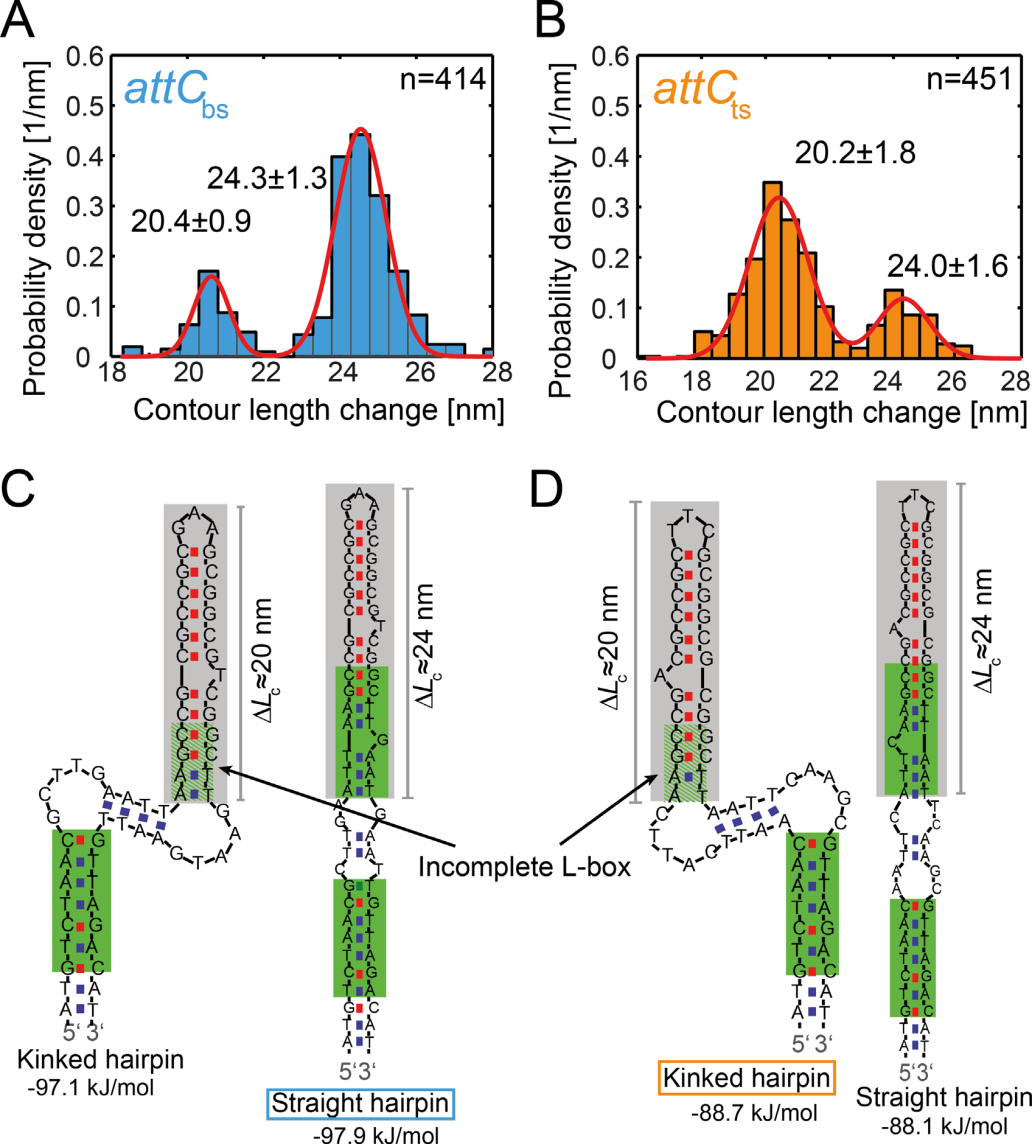
Contour length change of the major unfolding event. Histogram of the contour length change of the major unfolding and refolding event of the ${{att}}{{{C}}_{{\rm{bs}}}}$ and ${{att}}{{{C}}_{{\rm{ts}}}}$ in (**A**) and (**B**), respectively. UNAfold predictions (within 5% free energy difference) of the two most stable secondary structures with expected contour length changes of the upper stem for the ${{att}}{{{C}}_{{\rm{bs}}}}$ and ${{att}}{{{C}}_{{\rm{ts}}}}$ in (**C**) and (**D**), respectively. The upper stems with the expected contour length changes are indicated in grey. The thermodynamically preferred structure for the ${{att}}{{{C}}_{{\rm{bs}}}}$ is the straight hairpin (blue square) and for the ${{att}}{{{C}}_{{\rm{ts}}}}$ the kinked hairpin (orange square).

Interestingly, both ${{att}}{{{C}}_{{\rm{bs}}}}$ and ${{att}}{{{C}}_{{\rm{ts}}}}$ showed a broad contour length change of 20–25 nm (or 30–36 nt) upon the major unfolding event, which is unexpected for a well-folded DNA hairpin structure (19,21). Therefore, we analyzed the contour length distribution of *n* = 414 major unfolding and refolding events of ${{att}}{{{C}}_{{\rm{bs}}}}$. For the ${{att}}{{{C}}_{{\rm{bs}}}}$we found a bimodal distribution of contour length changes with one population (80%) showing a contour length change Δ*L*_maj,1_(${{att}}{{{C}}_{{\rm{bs}}}}$) = 24.3 ± 1.3 nm and a second population (20%) showing a contour length change Δ*L*_maj,2_(${{att}}{{{C}}_{{\rm{bs}}}}$) = 20.4 ± 0.9 nm (Figure 3A). These two contour length changes were also observed within a single tether (Supplementary Figure S3) and correspond to a release of 35 ± 2 nt and 30 ± 1 nt, respectively. In order to correlate the release of nucleotides with the structure of the DNA hairpin, we performed a DNA secondary structure prediction using UNAfold (17). Calculations for the full length ${{att}}{{{C}}_{{\rm{bs}}}}$ yielded the existence of two conformations with nearly identical folding free energies: a straight hairpin and a kinked hairpin structure (Figure 3C).

Considering that the ${{att}}{{{C}}_{{\rm{bs}}}}$ hairpin unfolds in a two-step process *via* a mechanically stable intermediate structure starting from the 5′- and 3′-ends, we hypothesize that it is the apical stems of the straight and kinked conformations (marked as grey areas, Figure 3C) that form two different mechanically stable intermediate states. For the kinked hairpin conformation, this intermediate state comprises 28 nt and for the straight hairpin 35 nt, both in excellent agreement with the measured 30 ± 1 nt and 35 ± 2 nt. Thus, we attributed the straight hairpin conformation to the population at ≈ 24 nm contour length change and the kinked conformation to the ≈ 20 nm contour length change. Remarkably, the larger population of ≈ 80% at Δ*L*_maj,1_(${{att}}{{{C}}_{{\rm{bs}}}}$) ≈ 24 nm correlates well with the lower free energy of the straight hairpin conformation predicted by the UNAfold calculation (Δ*G*_straight_(${{att}}{{{C}}_{{\rm{bs}}}}$) = –97.9 kJ/mol and Δ*G*_kinked_(${{att}}{{{C}}_{{\rm{bs}}}}$) = –97.1 kJ/mol). Therefore, we concluded that the experimentally observed bimodal distribution of the ${{att}}{{{C}}_{{\rm{bs}}}}$ contour length increase reveals the existence of two conformational states of which the straight hairpin is predominant.

The same analysis of the distribution of the contour length change was performed for the ${{att}}{{{C}}_{{\rm{ts}}}}$. A total of *n* = 451 major unfolding and refolding events of the ${{att}}{{{C}}_{{\rm{ts}}}}$ revealed again a bimodal distribution of contour length changes with a population Δ*L*_maj,1_(${{att}}{{{C}}_{{\rm{ts}}}}$) = 24.0 ± 1.6 nm (35 ± 2 nt, 24% of the events) and a second population at Δ*L*_maj,2_(${{att}}{{{C}}_{{\rm{ts}}}}$) = 20.2 ± 1.8 nm (30 ± 1 nt, 76% of the events, see Figure 3B). A UNAFold secondary structure prediction for ${{att}}{{{C}}_{{\rm{ts}}}}$ exhibited two energetically close conformations–again, a kinked hairpin and a straight hairpin (Figure 3D). In accordance with the ${{att}}{{{C}}_{{\rm{bs}}}}$, the upper stems comprised of 28 and 35 nt for the kinked and straight hairpins, respectively, and were assumed to represent the mechanically stable intermediate states for the ${{att}}{{{C}}_{{\rm{ts}}}}$ (grey highlight, Figure 3D). In contrast to the ${{att}}{{{C}}_{{\rm{bs}}}}$, for the ${{att}}{{{C}}_{{\rm{ts}}}}$, the occupancy of the ‘20 nm’ population, corresponding to the kinked hairpin conformation, is higher, which correlates well with the lower free energy of the kinked hairpin conformation of the ${{att}}{{{C}}_{{\rm{ts}}}}$ predicted by UNAfold (Δ*G*_straight_(${{att}}{{{C}}_{{\rm{ts}}}}$) = –88.1 kJ/mol and Δ*G*_kinked_(${{att}}{{{C}}_{{\rm{ts}}}}$) = –88.7 kJ/mol). Therefore, the predominant conformation for ${{att}}{{{C}}_{{\rm{ts}}}}$ hairpin upon folding is a kinked hairpin conformation. Notably, the analyses of contour length changes upon the major unfolding events during the first pulling cycle after tether formation yielded similar bimodal distributions for both ${{att}}{{{C}}_{{\rm{bs}}}}$ and ${{att}}{{{C}}_{{\rm{ts}}}}$ (Supplementary Figure S3). This corroborates the preceding analysis and renders the existence of force-induced structural heterogeneity unlikely. We therefore concluded that despite their complementary sequence, ${{att}}{{{C}}_{{\rm{bs}}}}$ and ${{att}}{{{C}}_{{\rm{ts}}}}$ preferentially fold into different structures.

### The unpaired central spacer of the *attC* strands influences the hairpin secondary structure

**Figure 4.**
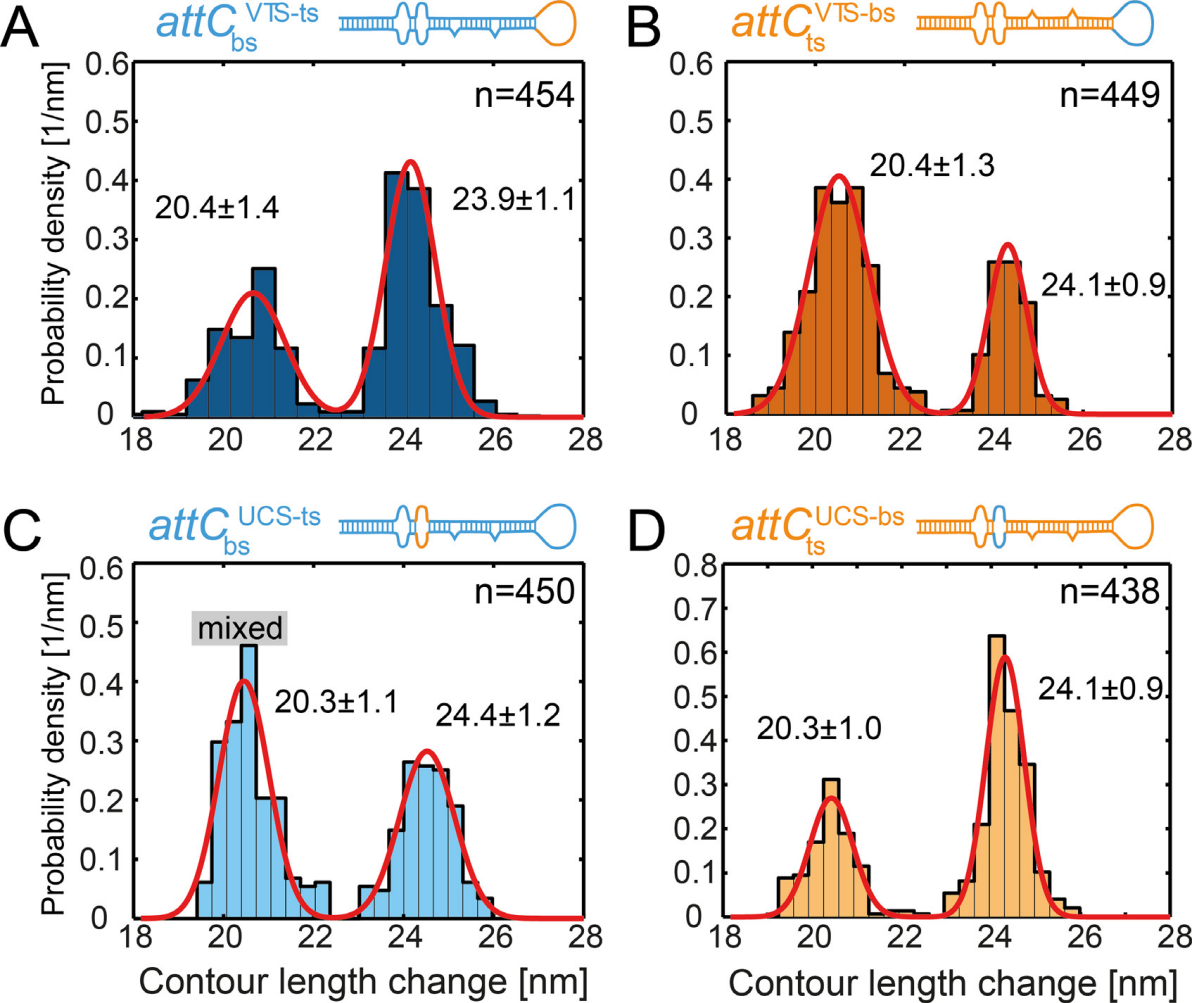
Contour length change of the major unfolding event of the attC mutants. Histograms of the contour length change of the major unfolding and refolding event of (**A**) ${{\ attC}}_{{\rm{bs}}}^{{\rm{VTS - ts}}}$, (**B**) ${{\ attC}}_{{\rm{ts}}}^{{\rm{VTS - bs}}}$, (**C**) ${{\ attC}}_{{\rm{bs}}}^{{\rm{UCS - ts}}}$ and (**D**) ${{\ attC}}_{{\rm{ts}}}^{{\rm{UCS - bs}}}$. Mutated regions in the hairpins are illustrated on top in color code (blue originally bottom strand, orange originally top strand). Predicted UNAfold structures are shown in Supplementary Figure S4.

As the bottom and top strands, although being complementary, assume different predominant conformations, the preference for a certain shape must be encoded in the hairpin sequence. We hypothesized that the structural elements–the variable terminal structure (VTS) and the unpaired central spacer (UCS) region–influence the thermodynamic stability and by that shift the equilibrium between the kinked and the straight hairpin. In order to test our hypothesis, we constructed hybrids of ${{att}}{{{C}}_{{\rm{bs}}}}$ and ${{att}}{{{C}}_{{\rm{ts}}}}$. In a first set of experiments, we exchanged the VTS sequence of the ${{att}}{{{C}}_{{\rm{bs}}}}$ with the VTS sequence of the ${{att}}{{{C}}_{{\rm{ts}}}}$ and vice versa to create ${{\ attC}}_{{\rm{bs}}}^{{\rm{VTS - ts}}}$ and ${{\ attC}}_{{\rm{ts}}}^{{\rm{VTS - bs}}}$, respectively (Supplementary Table S1B). Single-molecule force spectroscopy experiments on the hybrids revealed a similar two-step unfolding with an unfolding force of the major unfolding event of 6.5 ± 1.0 pN (*k* = 249, mean ± S.D.) for the ${{\ attC}}_{{\rm{bs}}}^{{\rm{VTS - ts}}}$ and 5.8 ± 0.8 pN (*k* = 241, mean ± S.D.) for the ${{\ attC}}_{{\rm{ts}}}^{{\rm{VTS - bs}}}$. Again, most of the traces showed only one, major unfolding event and only a few traces both the minor and the major unfolding events. For both ${{\ attC}}_{{\rm{bs}}}^{{\rm{VTS - ts}}}$ and ${{\ attC}}_{{\rm{ts}}}^{{\rm{VTS - bs}}}$ constructs, we observed a bimodal contour length change distribution, with the nearly-identical ratio between populations to the one observed for the wild-type ${{\ attC}}_{{\rm{bs}}}^{}$ and ${{\ attC}}_{{\rm{ts}}}^{}$, respectively (Figure 4A, B). UNAFold predicted for the hybrids ${{\ attC}}_{{\rm{bs}}}^{{\rm{VTS - ts}}}$ and ${{\ attC}}_{{\rm{ts}}}^{{\rm{VTS - bs}}}$ straight and kinked hairpins as possible conformations, with the straight hairpin being thermodynamically preferred for ${{\ attC}}_{{\rm{bs}}}^{{\rm{VTS - ts}}}$ and the kinked hairpin–for ${{\ attC}}_{{\rm{ts}}}^{{\rm{VTS - bs}}}$ (Supplementary Figure S5). UNAFold predictions were in excellent agreement with the results of our structural analysis (Figure 4A, B). It is important to note, that both the Δ*L*_c_ distributions and the predominant conformations for VTS-modified mutants were analogous to the corresponding ones for wild-type strands. Thus, we concluded that the VTS region does not determine the preferred conformation of the hairpin.

As the next step, we constructed ${{\ attC}}_{{\rm{bs}}}^{{\rm{UCS - ts}}}$and ${{\ attC}}_{{\rm{ts}}}^{{\rm{UCS - bs}}}$ hybrids by swapping 3 UCS bases between ${{\ attC}}_{{\rm{bs}}}^{}$ and ${{\ attC}}_{{\rm{ts}}}^{}$ (Supplementary Table S1B). Single-molecule optical tweezers experiments revealed the unfolding forces of 5.6 ± 0.8 pN (*k* = 229, mean ± S.D.) for ${{\ attC}}_{{\rm{bs}}}^{{\rm{UCS - ts}}}$ and 6.2 ± 0.8 pN (*k* = 212, mean ± S.D.) for the ${{\ attC}}_{{\rm{ts}}}^{{\rm{UCS - bs}}}$. Again, we observed two step unfolding patterns for both hybrids, which were similar to the wild-type ${{\ attC}}_{{\rm{bs}}}^{}$ and ${{\ attC}}_{{\rm{ts}}}^{}$. Both hybrids,${{\ attC}}_{{\rm{bs}}}^{{\rm{UCS - ts}}}$ and${{\ attC}}_{{\rm{ts}}}^{{\rm{UCS - bs}}}$, showed a bimodal distribution of contour length changes of their major unfolding event (Figure 4C, D). Surprisingly, the populations of the distributions were inverted in comparison to the wild-type strands (see Figure 3A, B). We correlated this change with structural predictions by UNAfold and found that for the ${{\ attC}}_{{\rm{ts}}}^{{\rm{UCS - bs}}}$ the energetically preferred conformation is a straight hairpin (in contrast to the kinked hairpin for$\ {{\ attC}}_{{\rm{ts}}}^{}$, Supplementary Figure S5). The UNAFold calculations for ${{\ attC}}_{{\rm{bs}}}^{{\rm{UCS - ts}}}$ revealed three energetically-similar structural predictions—a kinked and a straight hairpin configuration, and a hairpin with less base pairing in the UCS region (‘big bubble’, Supplementary Figure S6). Both upper stems of the ‘kinked’ and ‘big-bubble’ conformations contain 28 nt which corresponds to the contour length change of ≈ 20 nm. Thus, the enlarged population of ${{\ attC}}_{{\rm{bs}}}^{{\rm{UCS - ts}}}$ at ≈ 20 nm resembles a mixture of these two conformations (Figure 4C) with the prevailing ‘big-bubble’ sub-population over the ‘kinked’ according to the ratio of free energies (Supplementary Information). It is important to note, that the conformation of the hairpin with the big bubble is structurally closely related to the straight configuration. Indeed, e.g. two sides of the bubble could collapse on themselves turning a big-bubble hairpin into a straight one—a process which could occur during IntI binding (Supplementary Figure S7). The correlation of the mechanical structural analysis and the UNAfold calculations for UCS-hybrids sequences suggests that the UCS is an important regulator of the conformation of the *attC* DNA hairpin, and thus might be involved in the strand selectivity upon recombination.

### *In vivo* recombination is affected by a changed UCS sequence

**Figure 5.**
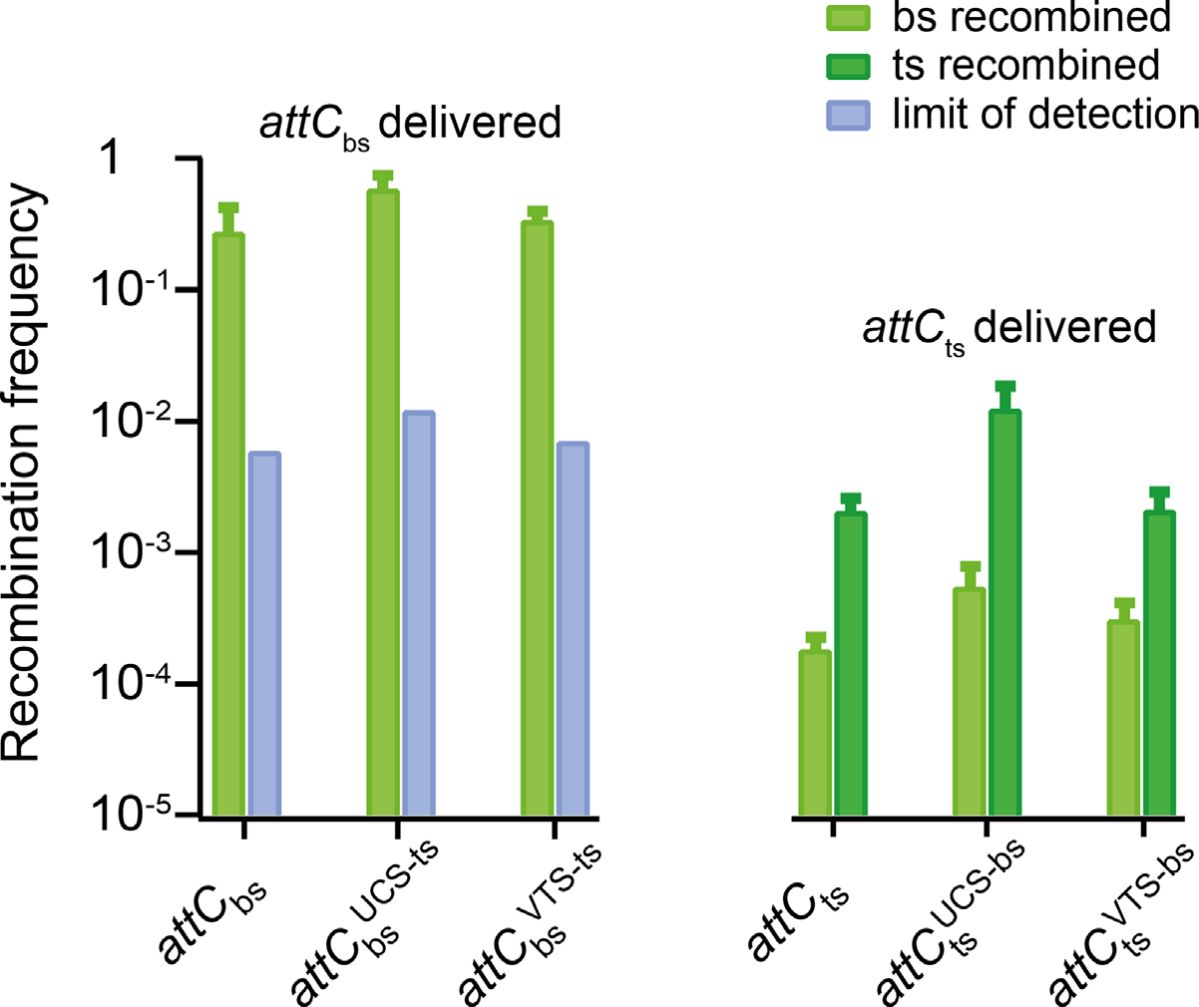
Recombination frequencies of wild-type and mutant *attC* sites *in vivo*. ${{\ attC}}_{{\rm{bs}}}^{}$ or ${{\ attC}}_{{\rm{ts}}}^{}$was delivered. Light green indicates bottom strand recombination, dark green indicates top strand recombination. Blue indicates the limit of detection (see Supplementary information). Values represent the mean of six independent experiments and error bars correspond to mean deviations.

Taking together the selectivity of IntI for the ${{att}}{{{C}}_{{\rm{bs}}}}$ during recombination (5–7) and the existence of different predominant conformations for the ${{att}}{{{C}}_{{\rm{bs}}}}$ and ${{att}}{{{C}}_{{\rm{ts}}}}$, we hypothesized that the strand selectivity of IntI is to a partially conditioned by the structural heterogeneity of the *attC* hairpin, e.g. the existence of kinked and straight conformations, which hinder or allow IntI binding, respectively. To test this hypothesis *in vivo*, we used a well-established assay (22) to characterize the recombination efficiency of wild-type ${{att}}{{{C}}_{{\rm{bs}}}}$ and ${{att}}{{{C}}_{{\rm{ts}}}}$, and the hybrid *attC* hairpins ${{\ attC}}_{{\rm{bs}}}^{{\rm{VTS - ts}}}$, ${{\ attC}}_{{\rm{ts}}}^{{\rm{VTS - bs}}}$, ${{\ attC}}_{{\rm{bs}}}^{{\rm{UCS - ts}}}$ and ${{\ attC}}_{{\rm{ts}}}^{{\rm{UCS - bs}}}$ (Figure 5).

As expected, for the ${{att}}{{{C}}_{{{aadA7}}}}$ site the delivery of the bottom strand lead to recombination at high efficiency (2.7 × 10^−1^, Figure 5, left panel, light green) and the recombination took place exclusively in the delivered bottom strand. The delivery of the top strand mostly led to recombination of *attC*_ts_ (Figure 5, right panel, deep green), even though we could also observe recombination of *attC*_bs_ (Figure 5, right panel, light green), which occurred after re-synthesis of the corresponding strand. However, the recombination frequency obtained in the case of top strand delivery was 100-fold lower than the one obtained upon bottom strand delivery (2.0 × 10^−3^ for ${{att}}{{{C}}_{{\rm{ts}}}}$*versus* 2.7 × 10^−1^ for ${{\ attC}}_{{\rm{bs}}}^{}$, Figure 5, ts and bs delivered), which originates from the lower affinity of IntI towards the ${{att}}{{{C}}_{{\rm{ts}}}}$ hairpin (5,6). The inversion of the VTS between ${{\ attC}}_{{\rm{bs}}}^{}$ and ${{\ attC}}_{{\rm{ts}}}^{}$ did not lead to any changes in recombination frequency and strand selectivity of IntI (Figure 5).

The mutation in the UCS region led to a 6-fold increase in recombination efficiency of ${{\ attC}}_{{\rm{ts}}}^{{\rm{UCS - bs}}}$ (1.21 × 10^−2^) in comparison to ${{\ attC}}_{{\rm{ts}}}^{}$ (2.03 × 10^−3^), which supports our hypothesis that a straight hairpin conformation assists the efficient recombination (Figure 5). The introduction of the top strand UCS in the ${{att}}{{{C}}_{{\rm{bs}}}}$ (${{\ attC}}_{{\rm{bs}}}^{{\rm{UCS - ts}}}$ mutant) did not notably affect the high recombination efficiency of the bottom strand (Figure 5) despite the inverted populations observed during optical-tweezers structural analysis. However, in this case we did not expect a significant drop in recombination efficiency, since the EHBs are major determinants of strand selectivity in *attC* sites, and their correct positioning in ${{att}}{{{C}}_{{\rm{bs}}}}$ has a predominant effect on IntI binding and recombination (Supplementary Figure S7) (5,6). Despite the fact that according to UNAfold calculations the preferred structure of ${{\ attC}}_{{\rm{bs}}}^{{\rm{UCS - ts}}}$ is the bubble-like hairpin that corresponds to a population with a short contour length increase upon unfolding, this structure is most likely recombinogenic due to its simple conversion to the straight hairpin upon IntI binding (Supplementary Figure S5).

Taken together, we showed that the exchange of three bases in UCS of one strand to the corresponding UCS bases of the opposite strand perturbs the conformational heterogeneity, favouring opposite conformations of the hairpin. By that, it may alter the *in vivo* recombination efficiency, as one (kinked) conformation exposes only one IntI binding box. The *in vivo* results support the hypothesis that the binding of IntI is influenced by the conformational heterogeneity of the *attC* hairpin. We conclude, that this intriguing mechanism of structural fine tuning is determined by the unpaired *attC* secondary structure elements–mainly by the UCS.

## DISCUSSION

### Low mechanical stability of imperfect DNA hairpin structures

DNA secondary structures such as hairpins are formed in the context of many biological processes (23). While they appear at first glance as simple structures consisting of an inverted repeat sequence, numerous structural features of natural hairpins remain elusive. In this study, we analyzed the DNA hairpins formed by the *attC_aadA7_* site, which is involved in DNA recombination of the bacterial integron system. According to previous single-molecule mechanical studies, simple stem-loop structures typically unfold and refold as a two-state system (19,21,24) along a single pathway (25,26) and exhibit mechanical stability on the order of 10–13 pN (19,27). In contrast, ${{attC}}_{{\rm{bs}}}^{}$ and ${{\ attC}}_{{\rm{ts}}}^{}$ hairpins unfold at lower forces (<6 pN) via two consecutive events and show evidence of complex folding dynamics involving on-pathway intermediates. The partial unfolding of the hairpins into intermediate states (step 1, Figure 2E) occurred mostly at forces lower than the detection limit, similar to a recently reported case for the HIV-1 RNA hairpin with an unstable lower stem (28,29) or DNA hairpins with single base damages like 8-oxoguanine (30). The major unfolding event for *attC* hairpins was typically at forces around 5.5 pN. This drop in unfolding force from 10 pN (typical for a simple stem–loop structure) most likely originates from the imperfections in the *attC* hairpin—namely, extra-helical bases and the unpaired central spacer, which prevent perfect base stacking and, by extension, high mechanical resistance. Such a low unfolding force may have functional implications like allowing an easy opening of DNA hairpins *in vivo* by polymerases or SSB (31,32) to minimize negative effects of unintended hairpin formation, e.g. during RNA transcription or DNA replication.

### ${{attC}}_{{\rm{bs}}}^{}$ and ${{\ attC}}_{{\rm{ts}}}^{}$ show distinct conformations

Analysing the contour length changes in detail allowed us to find a heterogeneous ensemble of two conformations for both ${{attC}}_{{\rm{bs}}}^{}$ and ${{\ attC}}_{{\rm{ts}}}^{}$. We correlated the measured contour length changes with secondary structure predictions by UNAfold to identify a kinked hairpin structure in addition to the canonical straight DNA hairpin. While secondary structure heterogeneity for nucleic acids is long known, here using our optical tweezers assay we directly observed two competing structures. Importantly, both structures are energetically very similar and differ less than 1 kJ/mol (≈0.4 *k*_B_T) in their predicted stability, which allowed direct observation of a population of both structural states. We further revealed that, the ${{attC}}_{{\rm{bs}}}^{}$ predominantly adopts a straight-hairpin structure, while the ${{attC}}_{{\rm{ts}}}^{}$ favours a kinked hairpin in about 75% of the analysed molecules. In Table 1 we compare the experimental ratio of straight canonical *vs* kinked structures. Notably, a discrepancy in the absolute numbers exist between the experimentally observed populations and those expected according to the Boltzmann distribution based on the folding free energies. This could indicate that in our experimental test, the refolded structures are biased by the remaining folding force. We therefore analysed also the first unfolding events of DNA tethers and observed a similar ratio as in our cumulative distribution. Thus, we assume that the refolding under external force does not skew the structural distributions. This might indicate that the calculations of the folding free energies are still lacking some unknown contributions. It is important to note, that already minor contributions of 1 kJ/mol or less affect the populations.

**Table 1.** Summary of the experimentally determined contour length changes and populations as well as the predictions (grey shading) based on UNAfold calculations

		Measured *L*_maj_, nm (nt) occurrence/occurrence in first unfolding		Predicted *L*_maj_, nm (nt) Predicted occurrence	
	# of unfolding-refolding events *n*/# of tethers *m*	Kinked hairpin conformation	Straight hairpin conformation	Kinked hairpin conformation	Straight hairpin conformation
${{\ attC}}_{{\rm{bs}}}^{}$	*n* = 414/*m* = 65	20.4±0.9 nm (∼30 nt)	24.3±1.3 nm (∼35 nt)	19 nm (28 nt)	23.8 nm (35 nt)
		20%/15%	80%/85%	42%	58%
${{\ attC}}_{{\rm{ts}}}^{}$	*n* = 451/*m* = 65	20.2±1.8 nm (∼30 nt)	24.0±1.6 nm (∼35 nt)	19 nm (28 nt)	23.8 nm (35 nt)
		76%/83%	24%/17%	56%	44%
${{\ attC}}_{{\rm{bs}}}^{{\rm{VTS - ts}}}$	*n* = 454/*m* = 41	20.4±1.4 nm (∼30 nt)	23.9±1.1 nm (∼35 nt)	19 nm (28 nt)	23.8 nm (35 nt)
		38%/27%	62%/73%	42%	58%
${{\ attC}}_{{\rm{ts}}}^{{\rm{VTS - bs}}}$	*n* = 449/*m* = 29	20.4±1.3 nm (∼30 nt)	24.1±0.9 nm (∼35 nt)	19 nm (28 nt)	23.8 nm (35 nt)
		69%/59%	31%/41%	56%	44%
${{\ attC}}_{{\rm{bs}}}^{{\rm{UCS - ts}}}$	*n* = 450/*m* = 29	20.3±1.1 nm (∼30 nt)	24.4±1.2 nm (∼35 nt)	19 nm (28 nt)	23.8 nm (35 nt) 33%
		55%/41%	45%/59%	14%	bubble: 19nm (28nt) 53%
${{\ attC}}_{{\rm{ts}}}^{{\rm{UCS - bs}}}$	*n* = 438/*m* = 30	20.3±1.0 nm (∼30 nt)	24.1±0.9 nm (∼35 nt)	19 nm (28 nt)	23.8 nm (35 nt)
		33%/10%	67%/90%	33%	67%

Based on the correlation of the recombination efficiency with a preferred secondary structure, we speculate that due to the absence of one complete IntI binding box, it is likely that the kinked conformation would lead to a significantly reduced IntI binding and, by extension, contribute to strand selectivity. Indeed, an earlier study already reported a correlation between a calculated probability to form a canonical straight hairpin structure and the measured recombination efficiency (8). Here, we observed directly the duality of a straight canonical structure and a kinked structure for the same *attC* site. We speculate that not only recognition of the kinked hairpin structure by IntI could be impaired, but also the recombination due to a structural offset, which would disrupt the interaction site between both integrase monomers or generate a steric hindrance during synaptic complex formation during *attC* × *attC* or *attC* × *attI* recombination.

### The conformational switch can be induced by three bases

Previous studies reported that a switch of the entire UCS of the ${{\ attC}}_{{\rm{bs}}}^{}$ to the ${{\ attC}}_{{\rm{ts}}}^{}$ can increase the recombination efficiency of the ${{\ attC}}_{{\rm{ts}}}^{}$ (5,6). Here, we found that changes in the UCS affect the structure adopted by the *attC* site significantly. The exchange of only three nucleotides in the UCS region inverts the populations of kinked and straight conformations (Figure 4C, D). Indeed, ${{\ attC}}_{{\rm{ts}}}^{{\rm{UCS - bs}}}$ adopted a canonical straight hairpin and showed a 6-fold increase in recombination efficiency. This effect of three exchanged nucleotides can be explained by analysing the free energy of the loop formed by these nucleotides based on a previous model (33). This model considers an energetic penalty of a mismatched base pair next to a Watson-Crick base pair, i.e. loss of base stacking interactions. The purine-containing mismatches show in calculations a lower energetic penalty (${{attC}}_{{\rm{bs}}}^{}$ and ${{\ attC}}_{{\rm{ts}}}^{{\rm{UCS - bs}}}$) than the pyrimidine-containing mismatches (${{\ attC}}_{{\rm{ts}}}^{}$ and ${{attC}}_{{\rm{bs}}}^{{\rm{UCS - ts}}}$). Thus, the purine-containing UCS region (A, G) is more stabilized than the pyrimidine one (C, T). Noteworthy, changes in the VTS did neither affect the structural ensemble compared to the wild-type nor the recombination efficiency. However, the VTS of the *attC_aadA7_* site contains only a very small 3 nucleotide-long loop and most likely exerts only little influence on the structure formation process. Longer loops or even extended complicated VTS sequences–as present in e.g. the VCR_2/1_ site–might show a stronger contribution to a multidimensional conformational space (8).

### Straight and kinked conformations in *attC* sites

Is the observed conformational preference to straight and kinked structures a common structural motif in *attC* sites? To answer this question, we performed a bioinformatics analysis of 263 *attC* sites provided by the authors of the INTEGRALL database (18). We predicted the structures of these *attC* sites using UNAfold and often found multiple structural models within the interval of 5% folding energy difference. We classified these models in kinked or straight conformations upon IntI binding site pairing (see Materials and Methods). Using a Boltzmann equation, we calculated the probabilities of conformational occupancy and found that the *attC* top strands are 30% less likely to form a canonical straight conformation than *attC* bottom strands.

In summary, using a single-molecule optical tweezers assay we found a heterogeneous ensemble of *attC* DNA hairpin conformations. We resolved a conformational bias of the *attC_aadA7_* bottom strand for the canonical straight stem-loop conformation, while the *attC_aadA7_* top strand was found to be structurally biased toward a kinked DNA structure. Key for the bias of the ${{\ attC}}_{{\rm{bs}}}^{}$ to the straight conformation is a fine-tuned sequence element, the unpaired central spacer. Upon a sequence inversion of only three nucleotides, the population of conformations was inverted and the recombination efficiency of the ${{\ attC}}_{{\rm{ts}}}^{}$ was increased 6-fold *in vivo*. This may originate from a steric hindrance for the integrase binding and synaptic complex formation during recombination imposed by the kinked conformation of the DNA hairpin. We anticipate that this fine structural regulation of conformations is also used in other DNA hairpin systems involved in DNA replication, transcription and recombination to ensure not only strand selectivity, but also stability and functionality.

## Supplementary Material

Supplementary Data
