# Supplementary material for: Structural heterogeneity of attC integron recombination sites revealed by optical tweezers
Source: Nucleic Acids Res. 2018 Dec 19;47(4):1861–70. doi: 10.1093/nar/gky1258 (PMC6393395; doi:10.1093/nar/gky1258)
Supplement: Supplementary Data [file gky1258_supplemental_files.pdf]

Supporting Information for

Structural heterogeneity of *attC* integron recombination site revealed by optical tweezers

Ann Mukhortava[1,‡], Matthias Pöge[1,2, ‡], Maj Svea Grieb[1], Aleksandra Nivina[3,4,5,6], Celine Loot[3,4], Didier Mazel[3,4] and Michael Schlierf*[1]

[1]B CUBE – Center for Molecular Bioengineering, TU Dresden, Tatzberg 41, 01307 Dresden

[2]Current Address: Max Planck Institute of Biochemistry, Am Klopferspitz 18, 82152 Martinsried, Germany

[3]Institut Pasteur, Unité de Plasticité du Génome Bactérien, Département Génomes et Génétique, 28 Rue du Dr Roux, 75015 Paris, France

[4]CNRS, UMR3525, 28 Rue du Dr Roux, 75015 Paris, France

[5]Paris Descartes University, 75006 Paris, France

[6]Current Address: Department of Chemistry, Stanford University, CA 94305, USA

‡ Equal contribution

* correspondence: michael.schlierf@tu-dresden.de

Supplementary Figures

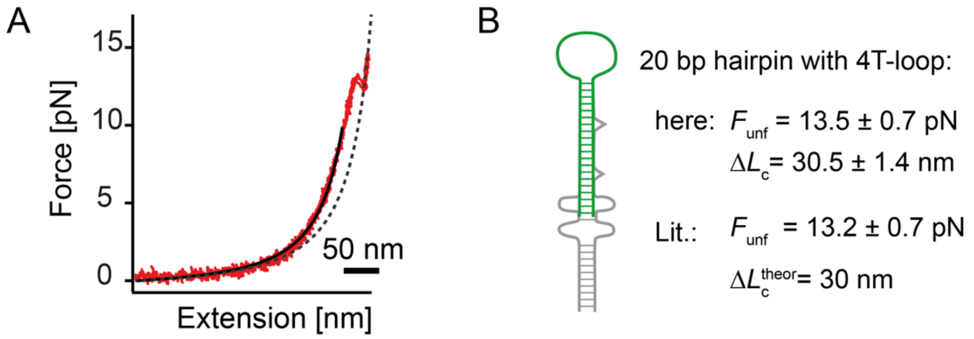

Supplementary Figure S1. Unfolding of the perfect hairpin 20R55/4T (sequence from (1)). **(A)** Typical force-extension curve (red) of the hairpin 20R55/4T, displaying WLC behavior of the handles at low force (solid black line) followed by hairpin unfolding at 13.5 ± 0.7 pN (*n*=10, mean ± s.d.). WLC fit to the contour length increase after hairpin unfolding (dotted black line) gives 30.5 ± 1.4 nm. The good agreement of the unfolding force obtained for that control sample with the literature value (13.2 ± 0.7 pN, ΔL_c^{theor} =30 nm (1)) illustrates the strong difference to the low unfolding force obtained for the $attC_{\mathrm{aadA7}}$hairpin. **(B)** 20R55/4T hairpin (green) is overlaid with the bottom strand of the bottom strand of $attC_{aadA7}$ hairpin (grey) for the size comparison.

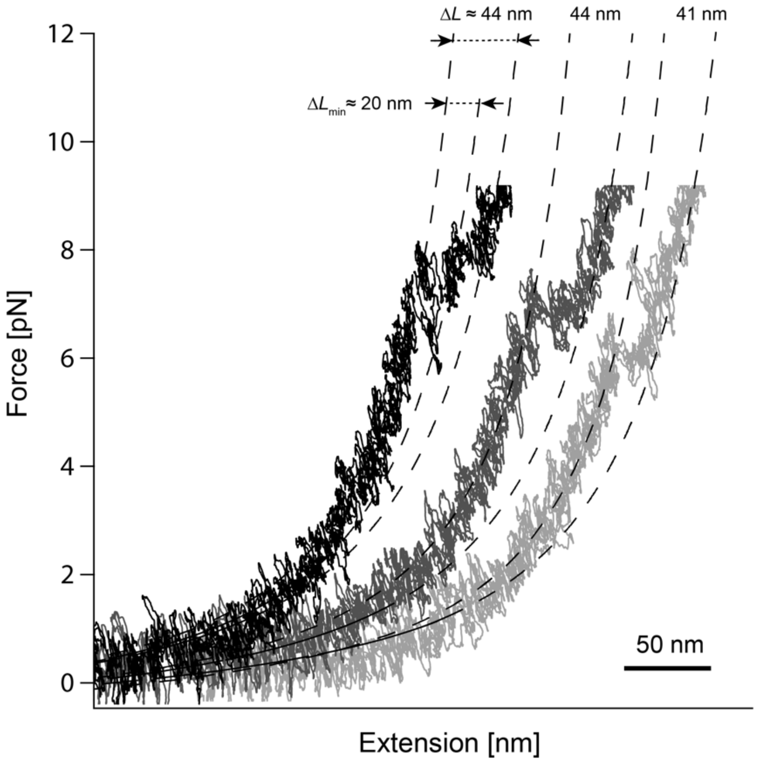

Supplementary Figure S2. Unfolding of the *attC*$_{bs}$ at in additional presence of 20 mM $MgCl_2$. Three typical unfolding force-extension traces of *attC*$_{bs.}$ Dashed lines show worm-like chain elasticity models. First trace shows a clear partial unfolding, other traces show only one total unfolding of the hairpin. Traces are offset for clarity.

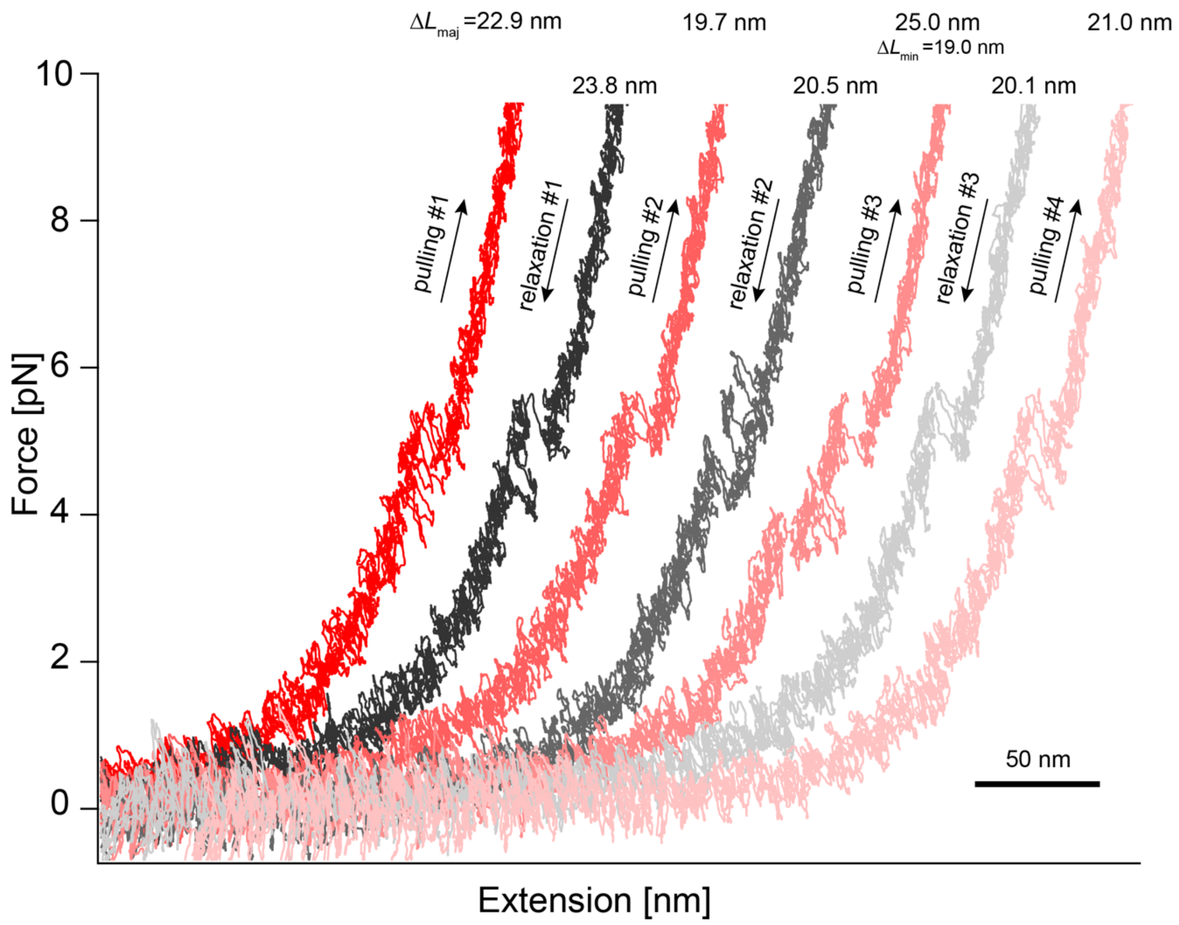

Supplementary Figure S3. Contour length changes of a single tether in multiple pulling and relaxation cycles. Typical example of a force-extension trace collection of a single tethered *attC*$_{bs}$ repetitively unfolded (red) and refolded (black). Refolding of the major event occurs at similar forces as unfolding indicating a close to equilibrium experiment. This is further supported by the multiple unfolding and refolding events in each cycle. Pulling cycle 3 shows a very stable minor unfolding event releasing a contour length of 19 nm that adds up with the major contour length change of 25 nm to a total unfolding of 44 nm, which is in good agreement with the expected 42 nm for the *attC*$_{bs}$ hairpin. Traces are offset for clarity.

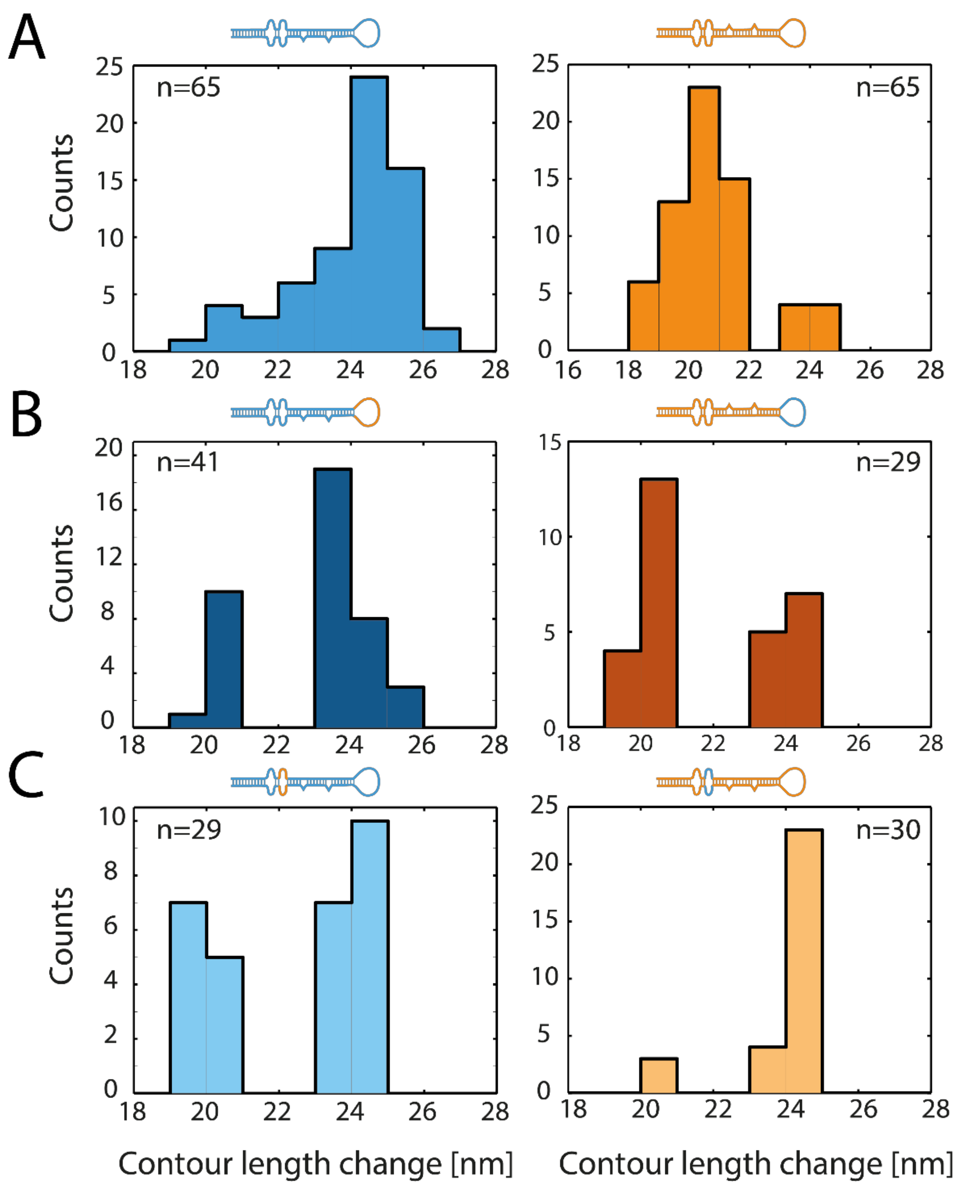

Supplementary Figure S4. Contour length increase upon major unfolding during the first pulling cycle. The bottom (left column) and top (right column) strand hairpins of wild-type *attC*$_{aadA7}$ hairpin and its mutants: **(A)** *attC*$_{\mathrm{bs}}$ and *attC*$_{\mathrm{ts}}$, **(B)** *attC*$_{\mathrm{bs}}^{\mathrm{VTS\text{-}ts}}$ and *attC*$_{\mathrm{ts}}^{\mathrm{VTS\text{-}bs}}$ and **(C)** *attC*$_{\mathrm{bs}}^{\mathrm{UCS\text{-}ts}}$ and *attC*$_{\mathrm{ts}}^{\mathrm{UCS\text{-}bs}}$. The "nascently"-folded, unperturbed DNA hairpins show bimodal distributions of contour length increase with the same trend in the ratio between 20- and 24-nm populations as the hairpins subjected to consecutive unfolding and refolding. Thus, conformational heterogeneity of hairpin intermediate states is most likely an intrinsic property of the hairpin itself and not force-induced.

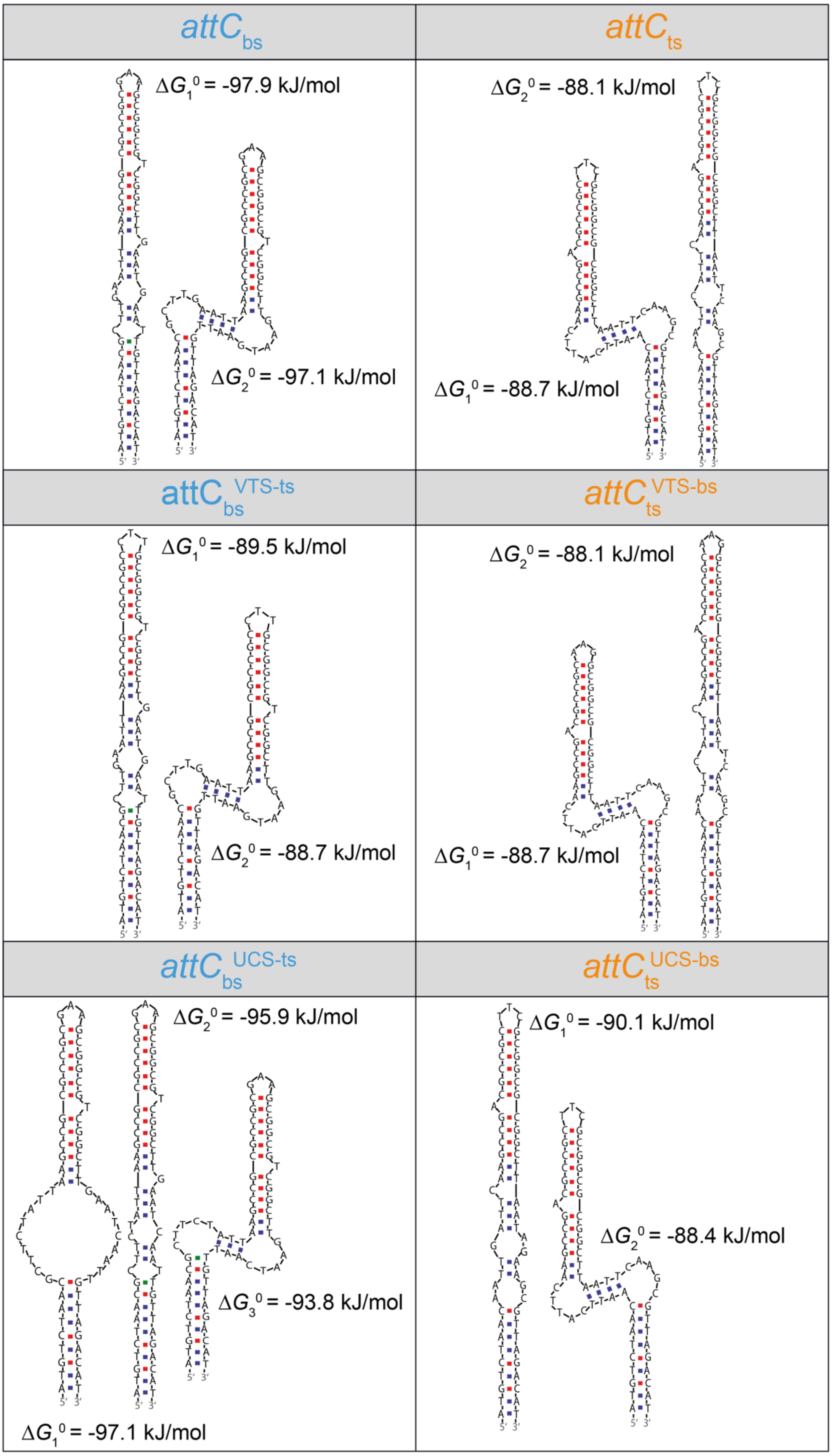

Supplementary Figure S5. UNAFold-predicted conformations and the corresponding free energies of hairpins formed by *attC*$_{\mathrm{aadA7}}$ wild-type and mutants.

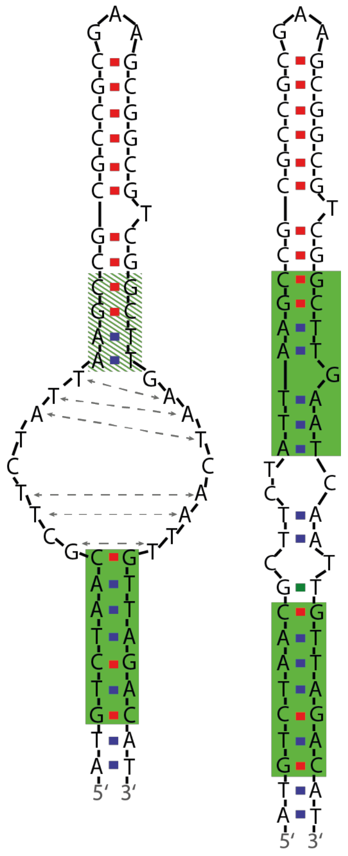

Supplementary Figure S6. Similarity between straight conformation and the big bubble conformation of the $\boldsymbol{attC_{bs}^{UCS\text{-}ts}}$ **hairpin.** Green areas represent IntI binding site. The filled boxes indicate completely folded binding boxes. The dashed box marks the paired bases of the partially folded binding L-box. The dashed lines show the possible coupling between bases of the bubble. Integrase binding supposedly collapses the bubble conformation into the rather conical and recombinogenic hairpin structure.

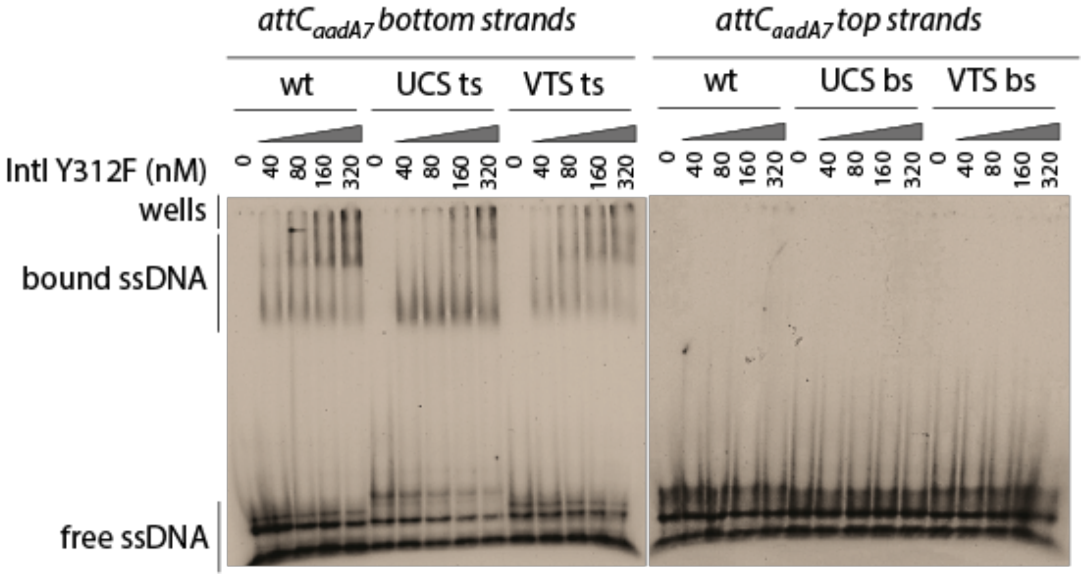

Supplementary Figure S7. Electrophoretic mobility shift assay of attC wild-type and attC mutants with IntI. The electrophoretic mobility shift assay of *attC*$_{aadA7}$ wild-type (bottom and top strands) and its mutants with IntI shows IntI binding to those sequences used in optical tweezers experiments. All ssDNA samples were run alone and after incubation with four increasing concentrations of IntI. The naked DNA bands are located at the lower end of the gel. IntI binding is characterized by a band shifted to the top of the gel. The bound fractions could correspond to single *attC* sites bound by one or two integrase monomers, or to synapses formed by several *attC* sites and integrases. As expected, the recombinogenic *attC* bottom-strand is bound by IntI for all concentrations tested, reflecting the high affinity of IntI to this site. The non-recombinogenic top-strand is not bound by IntI, justifying its use as a negative control for IntI binding. Both $attC_{\text{bs}}^{\text{VTS-ts}}$ and $attC_{\text{bs}}^{\text{UCS-ts}}$ show shifted bands for all IntI concentrations. For $attC_{\text{ts}}^{\text{UCS-bs}}$ the lack of IntI binding despite the dominant straight conformation can be explained by the steric hindering of integrase binding by EHBs, as reported in (2, 3).

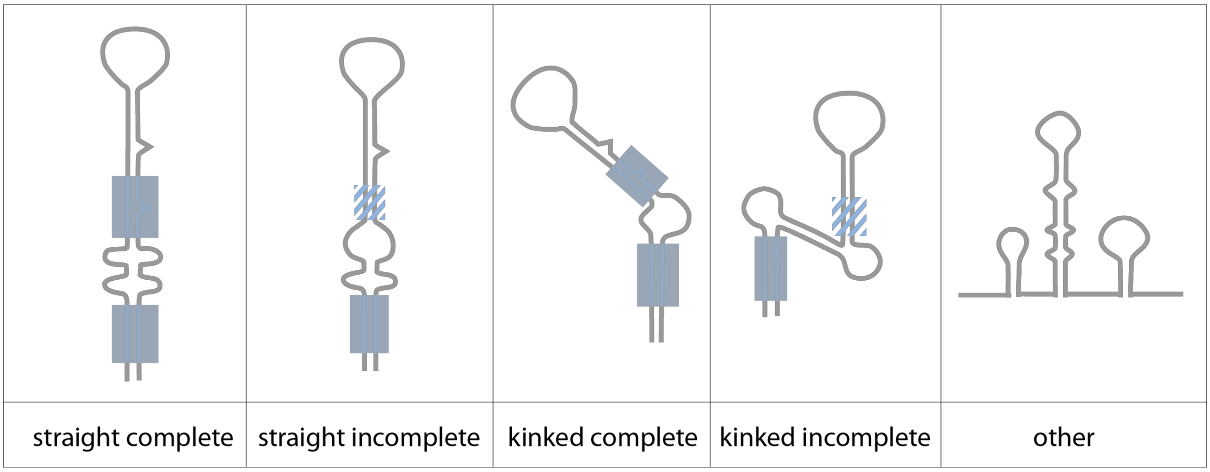

Supplementary Figure S8. Classification of attC sites from the INTEGRALL database conformations upon bioinformatics analysis: (1) straight-complete hairpins with R- and L-boxes fully paired, (2) straight-incomplete hairpins with paired R-box and not fully paired L-box; (3) kinked-complete hairpins with R- and L-boxes fully paired, (4) kinked-incomplete hairpins with paired R-box and not fully paired L-box and (5) other structures with fully unpaired R- and/or L-boxes.

Supplementary Table

Supplementary Table S1: Oligonucleotides used in this study. Sequences are given in 5’ → 3’ direction.

A. Primers used in PCR amplification of handles from lambda DNA for optical tweezers experiments. Underlined sequences are used as restriction sites.

Biotin handle	
Forward	Bio-CAGCATTGGTGACCTTGTTC
Reverse	GGTATAGCATTCTAGCATTCCATAGCTGATAACAATTGAGC
Digoxigenin handle	
Forward	Dig-ATCCGCAGAAGACGCAGATGCC
Reverse	ACGCAATGCGCAATGCAGCATACGCATGGCGGCTTTATAC

B. DNA oligomers used in the force spectroscopy experiments. Underlined sequences represent the hybridization part of the sample strand with the anchor strand. Additional 3T spacer in italic. For the $attC_{aadA7}$ mutants: exchanged fractions of the sequence from the bottom strand into the top strand and vice versa are marked in blue and orange, respectively. Sequences are given in 5’ → 3’ direction.

#	name	sequence
1	$attC_{bs}$	CATTGCGCATTGCGT*TTT*<br>ATGTCTAACGCTTGAATTAAGCCGCGCCGCGAAGCGGCGTCGGCTTGAATGAATTGTTAGACAT<br>*TTT*GGTATAGCATTCTAG
2	$attC_{ts}$	CATTGCGCATTGCGT*TTT*<br>ATGTCTAACAATTCATTCAAGCCGACGCCGCTTCGCGGCGCGGCTTAATTCAAGCGTTAGACAT<br>*TTT*GGTATAGCATTCTAG
3	$attC_{bs}^{VTS\text{-}ts}$	CATTGCGCATTGCGT*TTT*<br>ATGTCTAACGCTTGAATTAAGCCGCGCCGCCTTGCGGCGTCGGCTTGAATGAATTGTTAGACAT<br>*TTT*TGGTATAGCATTCTA
4	$attC_{ts}^{VTS\text{-}bs}$	CATTGCGCATTGCGT*TTT*<br>ATGTCTAACAATTCATTCAAGCCGACGCCGCAAGGCGGCGCGGCTTAATTCAAGCGTTAGACAT<br>*TTT*TGGTATAGCATTCTA
5	$attC_{bs}^{UCS\text{-}ts}$	CATTGCGCATTGCGT*TTT*<br>ATGTCTAACGCTTCTATTAAGCCGCGCCGCGAAGCGGCGTCGGCTTGAATCAATTGTTAGACAT<br>*TTT*TGGTATAGCATTCTA
6	$attC_{ts}^{UCS\text{-}bs}$	CATTGCGCATTGCGT*TTT*<br>ATGTCTAACAATTGATTCAAGCCGACGCCGCTTCGCGGCGCGGCTTAATAGAAGCGTTAGACAT<br>*TTT*TGGTATAGCATTCTA

C. DNA nucleotides used in in the Electrophoretic Mobility Shift Assays (EMSA).

#	name	sequence
1	$attC_{bs}$	ATGTCTAACGCTTGAATTAAGCCGCGCCGCGAAGCGGCGTCGGCTTGAATGAATTGTTAGACAT
2	$attC_{ts}$	ATGTCTAACAATTCATTCAAGCCGACGCCGCTTCGCGGCGCGGCTTAATTCAAGCGTTAGACAT
3	$attC_{bs}^{VTS\text{-}ts}$	ATGTCTAACGCTTGAATTAAGCCGCGCCGCCTTGCGGCGTCGGCTTGAATGAATTGTTAGACAT
4	$attC_{ts}^{VTS\text{-}bs}$	ATGTCTAACAATTCATTCAAGCCGACGCCGCAAGGCGGCGCGGCTTAATTCAAGCGTTAGACAT
5	$attC_{bs}^{UCS\text{-}ts}$	ATGTCTAACGCTTCTATTAAGCCGCGCCGCGAAGCGGCGTCGGCTTGAATCAATTGTTAGACAT
6	$attC_{ts}^{UCS\text{-}bs}$	ATGTCTAACAATTGATTCAAGCCGACGCCGCTTCGCGGCGCGGCTTAATAGAAGCGTTAGACAT

D. DNA nucleotides used to generate *attC* sites for the in vivo recombination assay, and for determining the recombined strand.

name		sequence
$attC_{aadA7}$ wt	Fw	AATTCATGTCTAACAATTCATTCAAGCCGACGCCGCTTCGCGGCGCGGCTTAATTCAAGCG TTAGACATG
	Rev	GATCCATGTCTAACGCTTGAATTAAGCCGCGCCGCGAAGCGGCGTCGGCTTGAATGAATTG TTAGACATG
$attC_{aadA7}$ UCSmut	Fw	AATTCATGTCTAACAATTGATTCAAGCCGACGCCGCTTCGCGGCGCGGCTTAATAGAAGCG TTAGACATG
	Rev	GATCCATGTCTAACGCTTCTATTAAGCCGCGCCGCGAAGCGGCGTCGGCTTGAATCAATTG TTAGACATG
$attC_{aadA7}$ VTSmut	Fw	AATTCATGTCTAACAATTCATTCAAGCCGACGCCGCAAGGCGGCGCGGCTTAATTCAAGCG TTAGACATG
	Rev	GATCCATGTCTAACGCTTGAATTAAGCCGCGCCGCCTTGCGGCGTCGGCTTGAATGAATTG TTAGACATG
SWbegin		CCGTCACAGGTATTTATTCGGCG
SWend		CCTCACTAAAGGGAACAAAAGCTG
MFD		CGCCAGGGTTTTCCCAGTCAC

Supplementary Materials and Methods

Protocol for Electrophoretic Mobility Shift Assay (EMSA)

Each reaction contained 500 ng Poly[d(I-C)], 12 mM Hepes–NaOH pH 7.7, 12% glycerol, 4 mM Tris–HCl pH 8.0, 60 mM KCl, 1 mM EDTA, 0.06 mg/ml BSA, 1 mM DTT, 10% Tween 20, 0.01 nM specified ^{32}P-labeled DNA oligonucleotide (Supplementary Table S1C) and the specified quantities of purified MBP-tagged IntI1Y312F (mutant unable to cleave DNA (4)), in a final volume of 20 µl. The samples were incubated at 30 ºC for 10 min without the probe followed by 20 min with the probe, then loaded to a 5% native polyacrylamide gels with 0.5x TBE as running buffer. The gels were visualized using a Molecular Dynamics intensification screen and a Typhoon FLA 9500 laser scanner.

Supplementary References

1. Woodside, M.T., Behnke-Parks, W.M., Larizadeh, K., Travers, K., Herschlag, D. and Block, S.M. (2006) Nanomechanical measurements of the sequence-dependent folding landscapes of single nucleic acid hairpins. *Proc. Natl. Acad. Sci.*, **103**, 6190–6195.

2. Nivina, A., Escudero, J.A., Vit, C., Mazel, D. and Loot, C. (2016) Efficiency of integron cassette insertion in correct orientation is ensured by the interplay of the three unpaired features of attC recombination sites. *Nucleic Acids Res.*, **44**, 7792–803.

3. Bouvier, M., Ducos-Galand, M., Loot, C., Bikard, D. and Mazel, D. (2009) Structural Features of Single-Stranded Integron Cassette attC Sites and Their Role in Strand Selection. *PLoS Genet.*, **5**, e1000632.

4. Johansson, C., Kamali-Moghaddam, M. and Sundström, L. (2004) Integron integrase binds to bulged hairpin DNA. *Nucleic Acids Res.*, **32**, 4033–4043.
